# CD13 as a new tumor target for antibody-drug conjugates: validation with the conjugate MI130110

**DOI:** 10.1186/s13045-020-00865-7

**Published:** 2020-04-07

**Authors:** Juan Manuel Domínguez, Gema Pérez-Chacón, María José Guillén, María José Muñoz-Alonso, Beatriz Somovilla-Crespo, Danay Cibrián, Bárbara Acosta-Iborra, Magdalena Adrados, Cecilia Muñoz-Calleja, Carmen Cuevas, Francisco Sánchez-Madrid, Pablo Avilés, Juan M. Zapata

**Affiliations:** 1 Research Department, PharmaMar S.A., Colmenar Viejo, Madrid, Spain; 2 Instituto de Investigaciones Biomedicas “Alberto Sols”, CSIC-UAM, Madrid, Spain; 3 Instituto de Investigacion Sanitaria La Paz, IdiPAZ, Madrid, Spain; 4 Department of Immunology, Instituto de Investigacion Sanitaria Hospital de la Princesa, IIS-IP, Madrid, Spain; 5 Centro Nacional de Investigaciones Cardiovasculares Carlos III, Madrid, Spain; 6 Department of Pathology, Instituto de Investigacion Sanitaria Hospital de la Princesa, IIS-IP, Madrid, Spain

**Keywords:** CD13, ADC, Antibody-drug conjugate, MI130110, Fibrosarcoma, Endocytosis, Aminopeptidase-N

## Abstract

**Background:**

In the search for novel antibody-drug conjugates (ADCs) with therapeutic potential, it is imperative to identify novel targets to direct the antibody moiety. CD13 seems an attractive ADC target as it shows a differential pattern of expression in a variety of tumors and cell lines and it is internalized upon engagement with a suitable monoclonal antibody. PM050489 is a marine cytotoxic compound tightly binding tubulin and impairing microtubule dynamics which is currently undergoing clinical trials for solid tumors.

**Methods:**

Anti-CD13 monoclonal antibody (mAb) TEA1/8 has been used to prepare a novel ADC, MI130110, by conjugation to the marine compound PM050489. In vitro and in vivo experiments have been carried out to demonstrate the activity and specificity of MI130110.

**Results:**

CD13 is readily internalized upon TEA1/8 mAb binding, and the conjugation with PM050489 did not have any effect on the binding or the internalization of the antibody. MI130110 showed remarkable activity and selectivity in vitro on CD13-expressing tumor cells causing the same effects than those described for PM050489, including cell cycle arrest at G2, mitosis with disarrayed and often multipolar spindles consistent with an arrest at metaphase, and induction of cell death. In contrast, none of these toxic effects were observed in CD13-null cell lines incubated with MI130110. Furthermore, in vivo studies showed that MI130110 exhibited excellent antitumor activity in a CD13-positive fibrosarcoma xenograft murine model, with total remissions in a significant number of the treated animals. Mitotic catastrophes, typical of the payload mechanism of action, were also observed in the tumor cells isolated from mice treated with MI130110. In contrast, MI130110 failed to show any activity in a xenograft mouse model of myeloma cells not expressing CD13, thereby corroborating the selectivity of the ADC to its target and its stability in circulation.

**Conclusion:**

Our results show that MI130110 ADC combines the antitumor potential of the PM050489 payload with the selectivity of the TEA1/8 monoclonal anti-CD13 antibody and confirm the correct intracellular processing of the ADC. These results demonstrate the suitability of CD13 as a novel ADC target and the effectiveness of MI130110 as a promising antitumor therapeutic agent.

## Introduction

CD13, also known as aminopeptidase-N (APN) and alanyl aminopeptidase (ANPEP) (EC 3.4.11.2; UniProt P15144), is a metallopeptidase originally described as a myeloid-specific hematopoietic marker [1]. It is a moonlighting ectoenzyme engaged in a wide range of biological functions (reviewed in [2]), most notably being involved in the post-secretory processing of secreted signaling peptides, regulating their access to cellular receptors. There are a number of results supporting the role of CD13 in tumor growth and metastasis [3, 4] as well as in angiogenesis [5, 6]. CD13 has been shown to be expressed in vessels of most neoplastic tissues as well as in tumor stroma [7]. Consistent with this, CD13 deficiency hampers tumor vascularization [4]. In addition, there is evidence showing a critical role of CD13-positive bone marrow-derived myeloid cells in supporting tumor growth, angiogenesis, and metastasis [8], thus highlighting CD13 as a potential antitumor target [9]. Besides, high expression of CD13 in cancer cells is associated with bad prognosis and poor patient survival in pancreas [10] and colon cancers [11], non-small cell lung cancer [12, 13], malignant pleural mesothelioma [14], hepatoblastoma [15], and soft tissue sarcoma [16] among others. In addition, CD13 has been shown to be a target for myeloid malignancies [17].

Antibody-drug conjugates (ADCs) are a class of therapeutic entities whose relevance in cancer treatment is endorsed by the successful cases of brentuximab vedotin and trastuzumab emtansine (Adcetris and Kadcyla, both regarded as remarkable milestones in the fight against Hodgkin’s lymphoma and breast cancer, respectively). New ADCs have been recently approved by the Food and Drug Administration (FDA) for the treatment of a variety of lymphoid malignancies, such as ozogamicin conjugates to gemtuzumab (Mylotarg), inotuzumab (Besponsa), approved by the FDA in 2017, and the most recent ADC examples moxetumomab pasudotox (Lumoxiti) and polatuzumab vedotin (Polivy), approved in 2018 and 2019, respectively (see [18, 19] for recent reviews). The successful cases of these ADCs have propelled efforts to discover and develop new conjugates as exemplified by the large number of clinical trials involving ADCs, exceeding 100 at the beginning of 2019 [20]. To expand the success of these four ADCs, it is imperative to identify novel antibody targets fulfilling the requirements needed for such a role: high expression on the tumor cell surface, differential expression in tumor versus normal cells, susceptibility to bind to a suitable antibody, and appropriate internalization rate as well as adequate intracellular trafficking [21]. In fact, some authors consider the antibody target as the most critical factor in the development of an active, therapeutically relevant ADC [22].

Considering the body of evidence described above and the fact that CD13 internalization can be achieved by using the CD13-binding Asn-Gly-Arg (NGR) tripeptide [4] and anti-CD13 monoclonal antibodies (mAb) [23, 24], CD13 may well be deemed as a suitable target for novel ADCs. However, it is not known whether the complex formed by CD13 and a suitable ADC would be efficiently internalized and processed intracellularly. We have recently published the successful use of PM050489, a marine molecule capable of binding tubulin at a novel site with nM affinity [25, 26], to prepare an ADC using a non-cleavable linker and the resulting conjugate MI130004 exhibited outstanding activity in several murine xenograft models for human tumors [27]. Prompted by this successful experience and urged by the curiosity to explore the potential of CD13 as a novel ADC target, we have conjugated PM050489 to a monoclonal anti-CD13 antibody and have investigated the biological effects of this ADC, called MI130110, in vitro as well as in in vivo models.

## Materials and methods

### Reagents

**Fig. 1:**
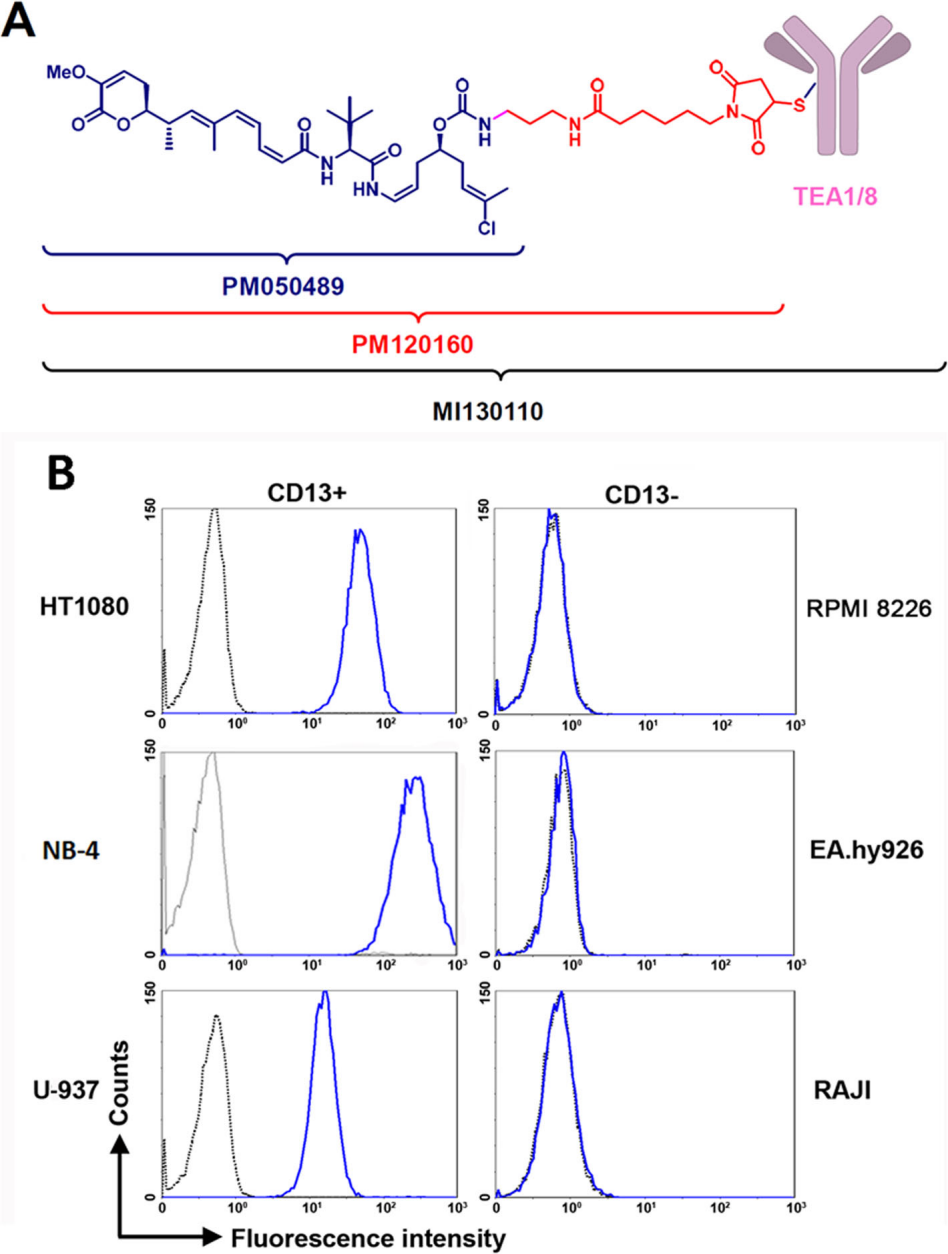
MI130110 structure and CD13 expression profile in cells. **a** Structure of MI130110, PM050489, and PM120160. **b** HT1080, NB-4, U-937, RPMI 8226, EA.hy926, and Raji cells (1E06) were incubated in the presence of TEA1/8 mAb (10 μg/mL) or the corresponding isotype control. After washing, cells were labeled with rabbit anti-mouse FITC and analyzed by flow cytometry

PM050489, PM120160 (the result of adding a non-cleavable linker to PM050489 as depicted in Fig. 1a, synthetic process described in [27]), and MI130110 were prepared in PharmaMar S.A. Chromatography reagents and materials were from GE Healthcare (Buckinghamshire, UK). Unless otherwise stated, reagents were purchased from Sigma-Aldrich (St Louis, MO). Given the null absorbance of PM120160 at 280 nm, antibody and ADC concentrations were determined spectrophotometrically by monitoring their absorbance at such wavelength using a molar extinction coefficient of 2.18E05 M^−1^ cm^-1^, a typical value for IgGs [28], and a molecular weight of 150 kDa.

### Preparation of the anti-CD13 TEA1/8 monoclonal antibody

The anti-CD13 TEA1/8 mAb was obtained in our laboratory using a hybridoma obtained from a fusion of SP2 mouse myeloma cells with splenocytes isolated from mice that had been immunized with human endothelial cells isolated from umbilical cord. TEA1/8 was clustered as an anti-CD13 antibody in the IV International Leukocyte Typing Workshop [29]. A detailed description of the methodology is provided in the supplementary information.

### Preparation and analysis of MI130110

A detailed description of the conjugation of PM120160 to TEA1/8 mAb is provided in Supplementary information. The resulting MI130110 ADC was purified by gel filtration in Sephadex G-25. ADC concentration was determined by spectrophotometry. Analysis of MI130110 by hydrophobic interactions chromatography (HIC) was performed on an Agilent 1100 HPLC system (Agilent, Santa Clara, CA) (see Supplementary information).

### Flow cytometry

For the analysis of CD13 expression, cells (1E07 cells/mL) were incubated in the presence of 10 μg/mL anti-CD13 TEA1/8 mAb or MI130110 or the corresponding isotype control for 30 min at 4 °C. After washing with cold phosphate buffered saline (PBS) at 400 g for 5 min, cells were incubated with rabbit anti-mouse-fluorescein isothiocyanate (FITC) antibody for 20 min at 4 °C, washed and analyzed by flow cytometry in a FACS Canto II cytofluorimeter (BD Biosciences). Cells from xenografted tumors were prepared as described in Supplementary information and analyzed by flow cytometry as described above.

To assess the internalization of the CD13 with either TEA1/8 mAb or MI130110, cells (1E07 cells/mL) were cultured in the presence of anti-CD13 TEA1/8 mAb or MI130110 or the corresponding isotype control at the indicated concentrations and times at 37 °C, in order to allow the endocytosis of the naked and conjugated anti-CD13 mAb. After washing with cold PBS at 40×*g* for 5 min, cells were labeled with rabbit anti-mouse FITC for 20 min at 4 °C. Then, cells were washed and analyzed by flow cytometry. Cells labeled with isotype control were used as negative control. The percentage of endocytosis was calculated as the decrease of mean fluorescence intensity (MFI) of CD13 staining after incubation at 37 °C relative to the CD13 MFI at 0 h.

### Cell cycle analysis

HT1080 and EA.hy926 cells (2E06 cells/mL) were cultured in the presence or in the absence of different concentrations of MI130110. After the indicated times, cells were harvested, fixed in ethanol, and stained with propidium iodide (PI) as previously described [30]. Cell cycle was analyzed by flow cytometry and the ModFit LT software (Verity Software House, Topsham, ME).

### Cell viability assay

A colorimetric assay based on the reduction of 3-(4,5-dimethylthiazol-2-yl)-2,5-diphenyltetrazolium bromide (MTT) was used for quantitative measurement of cell viability as already described [27].

### Cell death determination

Cells (1E05 per well) were cultured in 96-well microtiter plates in the presence or in the absence of increasing concentrations of MI130110. After 24, 48, and 72 h, cells were harvested and incubated with FITC-labeled annexin V (Immunostep, Salamanca, Spain) and 1 μg/mL PI (Sigma-Aldrich) in binding buffer (5 mM CaCl2, 10 mM Hepes, and 140 mM NaCl). After 15 min in the dark, they were analyzed by flow cytometry. Cell viability was measured as the percentage of annexin V and PI-double negative cells.

### Immunofluorescence

For fluorescence microscopy analyses HT1080, cells (1E05) were seeded onto poly-lysine-coated coverslips and cultured overnight at 37 °C and 5% CO_2_ atmosphere. Then, cells were incubated with 5 μg/mL of either anti-CD13 TEA1/8 mAb or MI130110 (IgG2a) and either kept on ice for at least 30 min or cultured at 37 °C for 3 h or 24 h to allow CD13 endocytosis, as indicated. For detection of CD13, cells were fixed with 1:1 (v/v) methanol/acetone, washed three times with cold PBS, and blocked with 10 mM Hepes, pH 7.4, 3% bovine serum albumin (BSA), and 100 μg/mL γ-globulin in PBS for 1 h at 37 °C. CD13 staining was achieved by incubating cells with Alexa 488-labeled rabbit anti-mouse antibodies for 1.5 h at 37 °C. For the analysis of mitosis, 0.75x1E5 HT1080 cells were fixed with neutral buffered 10% formalin solution for 10 min, washed and then blocked and permeabilized with buffer containing 10 mM Hepes, pH 7.4, 0.3% triton X-100, 3% BSA and 2% goat serum in PBS for 1 h at 37 °C. For mitosis analysis, cells were stained with anti-β-tubulin (IgG1, TUB2.1, Sigma) and either with α-tubulin (IgG2b, 66031-1-Ig, Proteintech) or with anti-acetylated α-tubulin (IgG2b, 6-11B-1, Sigma) in blocking buffer for 1.5 h at 37 °C. Then, cells were carefully washed with PBS 3 times and then incubated with anti-mouse IgG1-Alexa 488, anti-mouse IgG2b-Alexa 647, and with anti-mouse IgG2a-Alexa 594 (to detect endocyted CD13-complexes with either TEA1/8 or MI130110) in blocking buffer for 1.5 h at 37 °C. Nuclei and chromosomes were visualized by staining with 10 μg/mL of 4′,6-diamidino-2-phenylindole (DAPI) (Sigma-Aldrich) for 10 min at room temperature, and samples were then mounted on slides using Prolong (Thermo-Fisher Scientific). Confocal microscopy was performed using the Leica TCS SP5 Spectral Confocal Microscope system and the Zeiss LSM 710 confocal laser scanning microscope. Images were analyzed with the Image J software.

Nucleus staining of HT1080 tumors excised from mouse xenografts that were left untreated or treated with MI130110 for 24 h was performed by staining frozen slides of the tumors with a 1:5000 dilution in PBS of commercial Hoechst 33258 (Sigma-Aldrich).

### Xenograft murine models

Design, randomization, and monitoring of experiments (including body weights and tumor measurements) were performed using the NewLab Software v2.25.06.00 (NewLab Oncology, Vandoeuvre-Lès Nancy, France). Female athymic Nude-Foxn-1 nu/nu mice were used to generate xenografts of HT1080 whereas RPMI 8226 cells were xenografted in CB-17/IcrHsd-PrKdc-SCID mice, both mice strains supplied by Envigo, RMS Spain S.L. Animals between 4 to 6 weeks of age were subcutaneously xenografted with each cell into their right flank with circa 3-30 x 1E06 cells suspended in 0.05 ml of solution consisting of 50% Matrigel™ (Corning Inc., Corning, NY) and 50% cell culture medium without serum or antibiotics. When tumors reached circa 200 mm^3^, mice (*N* = 8–20 animals per group) were randomly allocated (Day 0) to receive the intended dose of MI130110, PM050489, anti-CD13 mAb, or vehicle. Intravenous treatments were weekly administered for 2 consecutive weeks. The control animals received an equal volume of vehicle with the same schedule. Caliper measurements of the tumor diameters were made three times a week, and tumor volumes were calculated according to the following formula: Volume = (*a* × *b*^2^)/2, where *a* and *b* were the longest and shortest tumor diameters, respectively. For survival evaluation, time to endpoint was defined as the time from day 0 to death as a result of tumor growth (larger than 2000 mm^3^) or any other cause (e.g., tumor necrosis). Complete tumor regression (CR) was defined as tumor volume below 63 mm^3^ for 2 or more consecutive measurements, such value corresponding to the lowest measurable limit considering the contribution of the mass from fibrous material, scar tissue, etc. Statistical differences, in animal survival, were assessed by Kaplan-Meier curves with the log rank test. Animals were humanely sacrificed when their tumors reached 2500 mm^3^ or if significant toxicity (e.g., severe body weight reduction) was observed. Differences in tumor volumes between treated and control group were evaluated using the Mann–Whitney *U*-test. Statistical analyses were performed by Graph Pad Prism® v5.03 (Graph Pad Software Inc. La Jolla, CA, USA).

## Results

### Cellular uptake of TEA1/8 and MI130110 upon interaction with CD13

Several human cell lines were examined to assess the expression levels of CD13 on their surface in order to select the most appropriate ones to perform our studies. As observed in Fig. 1b, flow cytometry data revealed that HT1080 (fibrosarcoma), U-937 (histiocytic lymphoma), and NB-4 (acute promyelocytic leukemia) cells showed high levels of CD13, whereas CD13 expression could not be detected in Raji (Burkitt’s lymphoma), RPMI 8226 (myeloma), and EA.hy926 (endothelium, non-tumor) cells. In addition to these immortalized cell lines, CD13 expression is found in normal tissues in cells from the myeloid lineage. Accordingly, CD13 levels were very prominent in some representative examples of acute myeloid leukemia and myeloid sarcoma (Suppl. Fig. 1 a-c). In addition, CD13 has been described to be expressed in a variety of tumors of distinct origin. Indeed, we show CD13 expression in a variety of tumors, including a sample of well-differentiated liposarcoma and another of dedifferentiated liposarcoma (Suppl. Fig. 1 d and e, respectively), a specimen of signet ring cell gastric adenocarcinoma (Suppl. Fig. 1f), and in two samples of ductal carcinoma (Suppl. Fig. 1 g and h). Interestingly, we have also observed CD13 expression on the endothelium of tumor blood vessels of a breast tumor sample with tumor cells lacking CD13 expression (Suppl. Fig. 1 i), confirming previous reports [7] and further stressing the potential of this protein as a possible tumor target.

**Fig. 2:**
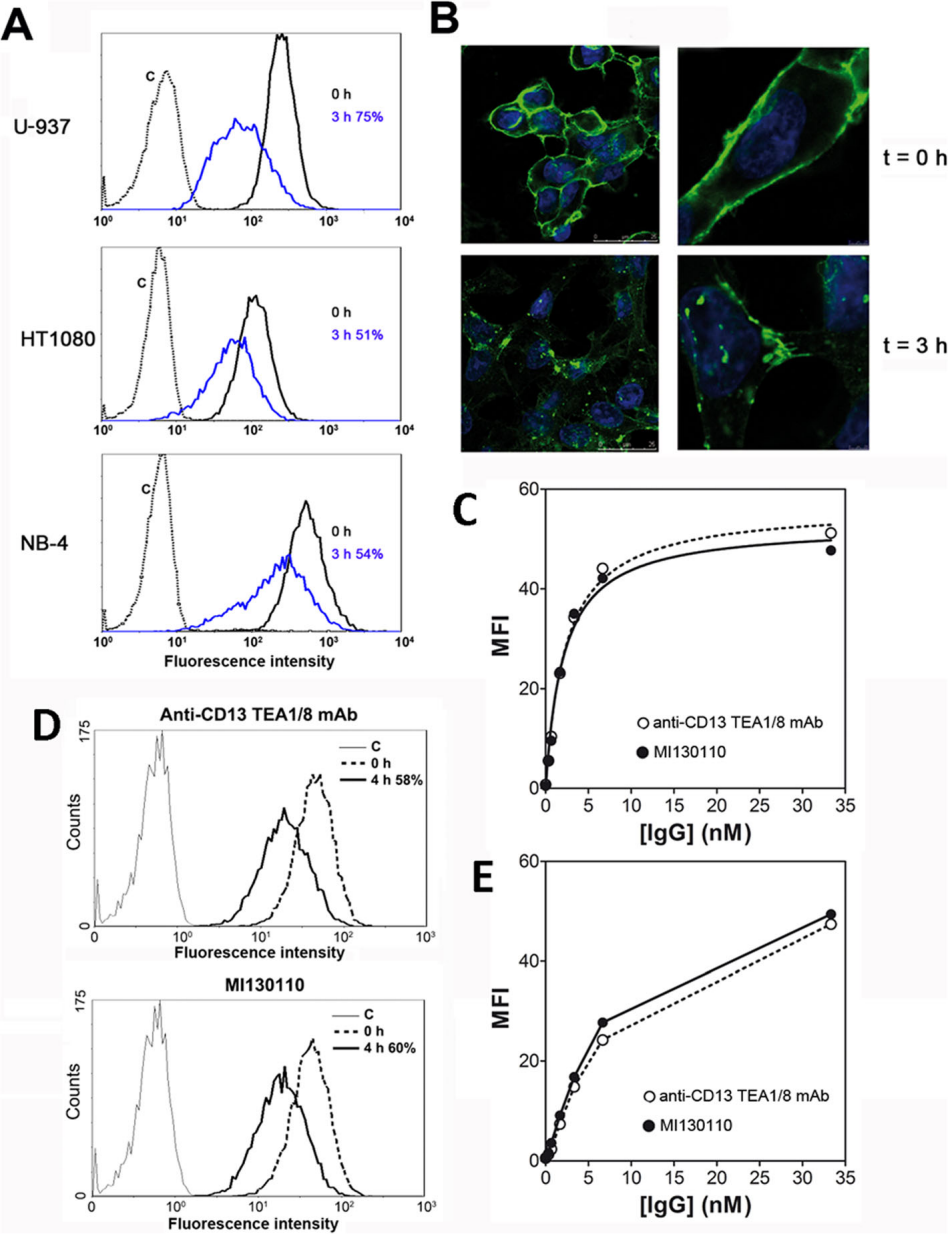
Cellular uptake of TEA1/8 and MI130110. **a** HT1080, U-937, and NB-4 cells (1E06) kept in suspension were incubated with TEA1/8 (10 μg/mL) for 3 h at 37 °C. Cells were then washed with cold PBS, labeled with rabbit anti-mouse FITC on ice for 30 min, and analyzed by flow cytometry. Cells labeled with isotype control were used as negative control. The percentage of CD13 endocytosis was calculated as the decrease of MFI of CD13 staining after 3 h incubation at 37 °C relative to the CD13 MFI at *t* = 0. **b** HT1080 cells (1E05) were plated in complete tissue culture medium onto poly-lysinated cover glasses and allowed to settle for 24 h at 37 °C and 5% CO_2_. Then, cells were left untreated or incubated with 5 μg/mL TEA1/8 for 3 h at 37 °C. Cells were fixed with 1:1 (v/v) methanol/acetone, washed, and labeled with rabbit anti-mouse FITC. Nuclei were visualized by DAPI staining. Figure shows untreated cells (*t* = 0), and cells allowed to internalize CD13 for 3 h (*t* = 3h). Samples were analyzed by confocal microscopy. Scale bars are shown. **c** HT1080 cells (1E06) were incubated with the indicated concentrations of TEA1/8 or MI130110 for 30 min on ice. After washing, cells were labeled with rabbit anti-mouse FITC and analyzed by flow cytometry. The resulting data were used to calculate the binding affinities of both molecules to CD13 by non-linear regression fitting of the experimental data to a classical binding isotherm equation considering one class of binding sites, the curves shown in the graph correspond to such regression. **d** HT1080 cells (1E06) were incubated in suspension with 10 μg/mL of either TEA1/8 or MI130110 at 37 °C for 4 h. Once washed, cells were labeled with rabbit anti-mouse FITC and analyzed by flow cytometry. Cells labeled with isotype control were used as negative control. The percentage of endocytosis at 4 h was calculated as the decrease of the MFI of CD13 staining after 4 h incubation at 37 °C relative to the CD13 MFI at *t* = 0. **e** HT1080 cells (1E06) were incubated in 200 μL culture medium with the indicated concentrations of TEA1/8 or MI130110 for 12 h at 37 °C to allow endocytosis to proceed. Then, 100 μL of the supernatants were harvested and used to stain CD13 on fresh HT1080 cells (1E06) as described for **c**. Concentrations in the horizontal axis correspond to those initially used in the first incubation

Next, the CD13-expressing cell lines U-937, HT1080, and NB-4 were then tested for their ability to internalize the TEA1/8-CD13 complex. Figure 2a shows a significant degree of internalization after a 3-h incubation of the cells with the antibody. Such cellular uptake can be quantified by measuring the decrease in MFI and accounted for a CD13 internalization of 75% for U-937, 51% for HT1080, and 54% for NB-4 of the total amount of CD13 normally expressed on the cell surface. The event was also visualized by fluorescence microscopy (Fig. 2b) with HT1080 cells: while at *t* = 0, most of the antibody remained bound to the plasma membrane, after 3 h a relevant portion of it can be detected as fluorescent spots within the cytosol, thus confirming that CD13 was indeed endocytosed and not lost by a proteolytic shedding mechanism.

The significant internalization rate of the antibody certainly endorses the suitability of CD13 as a possible ADC target, but it also led us to consider whether the conjugation of the antibody with PM050489 may affect such efficient uptake. The drug was conjugated to TEA1/8 as described under the “Methods” section and in the supplementary information (structure in Fig. 1a), and the performance of the conjugation was checked by HIC (Suppl. Fig. 2). Several peaks of higher hydrophobicity than that of the naked antibody (hence, suggesting the presence of several species of conjugates with different stoichoimetries) could be detected, confirming the success of the conjugation process. The new ADC, termed MI130110, was then tested for its ability to bind to CD13 in HT1080 cells and to promote the endocytosis of the antibody-antigen complex. According to Fig. 2c, both ADC and antibody bind to CD13 expressed on the surface of HT1080 cells with identical affinities (2.1 ± 0.3 nM for the ADC and 2.3 ± 0.3 nM for the antibody), thus demonstrating that the conjugation to PM050489 did not affect the ability of the antibody to bind its target. Notably, such binding reaches saturation at high concentrations, thus highlighting the specific nature of the binding. Moreover, flow cytometry experiments evidenced that MI130110 is readily endocytosed by HT1080 cells: 60% of the ADC is internalized in the HT1080 cells after 4 h incubation as calculated from the decrease of the MFI (Fig. 2d), a value similar to that rendered by the unconjugated antibody (58%) in the same experiment. This decrease in the antibody labeling on the cell surface is due to cellular uptake and not to spontaneous release to the milieu, as demonstrated above. The similar rate and efficiency of CD13 endocytosis induced by both anti-CD13 TEA1/8 mAb and MI130110 ADC is further confirmed by the similar amounts of both molecules remaining in the culture supernatant after 24 h of culture with HT1080 cells. Indeed, as shown in Fig. 2e, when the harvested supernatants are tested for binding to fresh HT1080, both TEA1/8 mAb and MI130110 supernatants produced identical binding curves although with lower amplitude compared to Fig. 2c, thus indicating that similar amounts of both unconjugated anti-CD13 mAb and MI130110 ADC were endocytosed by the cells.

### Biological effects of MI130110 in vitro

**Fig. 3:**
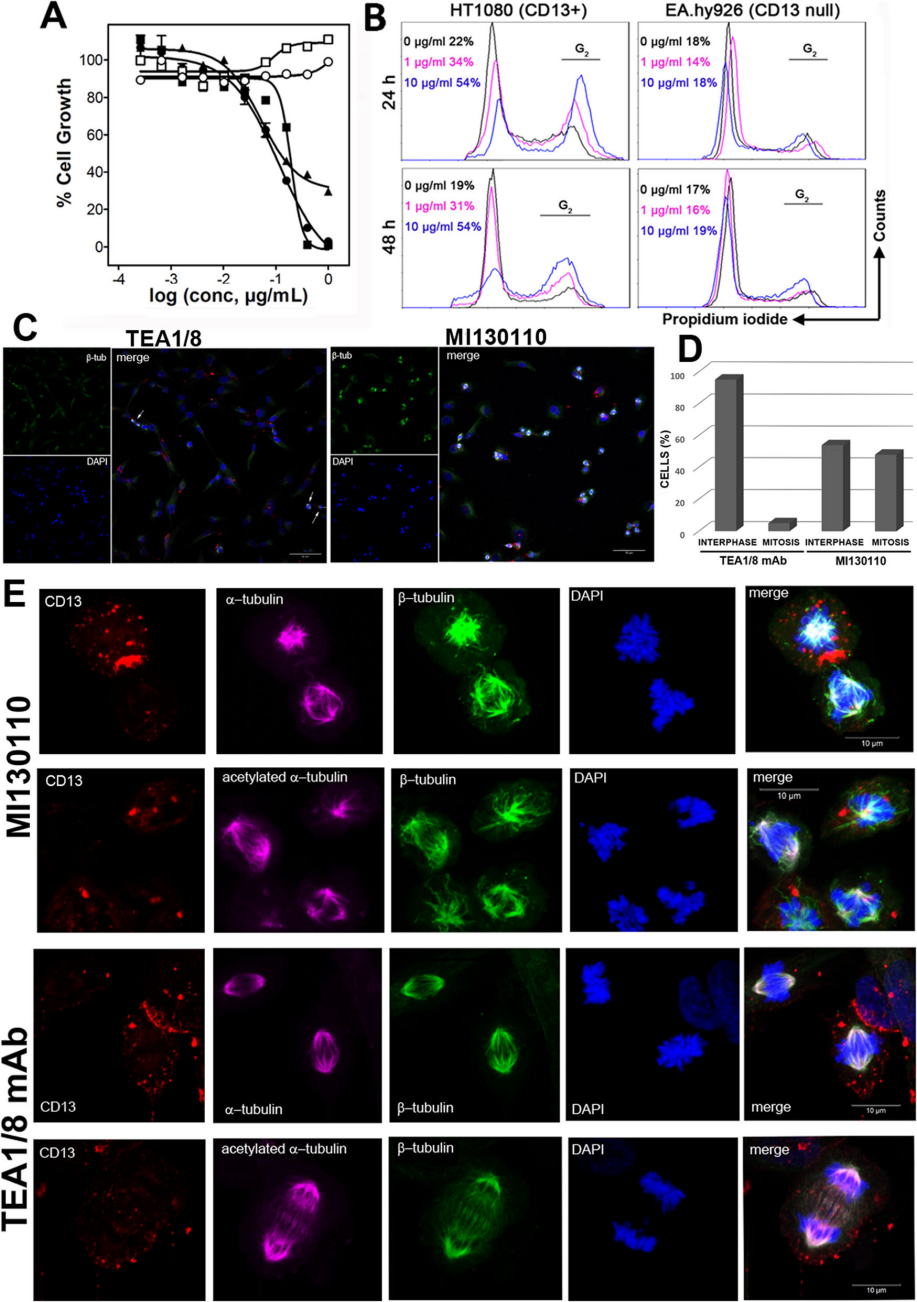
Effects of MI130110 on cell division. **a** Anti-proliferative assay showing the in vitro potency of MI130110. The assay was performed as described in the “Materials and methods” section with CD13-positive U-937 (solid circles), NB-4 (solid squares) and HT1080 (solid triangles) cell lines and CD13-negative RPMI 8226 (hollow circles) and Raji (hollow squares) cell lines. **b** HT1080 and EA.hy926 were cultured in the presence of different concentration of MI130110 (0, 1, and 10 μg/mL) for 24 and 48 h. Then, 2E06 cells were harvested, fixed in ethanol and their nuclei stained with PI, and analyzed by cytofluorimetry. The percentage values shown correspond to the percentage of cells in G2 phase. **c** HT1080 cells were incubated with either anti-CD13 TEA1/8 mAb (5 μg/mL) or MI130110 (5 μg/mL) for 24 h at 37 °C and processed as described in the “Materials and methods” section. The expression of β tubulin, DAPI-staining of DNA, and a merged composition of representative fields of cells treated with anti-CD13 mAb and ADC is shown. Scale bars are shown. **d** Quantification of cells in interphase and mitosis after 24 h treatment with anti-CD13 TEA1/8 mAb (5 μg/mL) or MI130110 (5 μg/mL). Cells were processed as described in **c**. Cells in interphase or undergoing mitosis from a total of 7 representative fields (24 x optical magnification) for each condition were counted. A total of 539 (93.5%) cells were in interphase and 20 (6.5%) in mitosis in the treatment with anti-CD13 TEA1/8 mAb. A total of 215 (54%) cells were in interphase and 185 (45%) in mitosis in the treatment with MI130110. **e** MI130110 treatment causes mitotic catastrophe. Cells were treated and processed for immunofluorescence as described in the “Materials and methods” section. Figure shows representative cells undergoing mitosis (128 x optical magnification) treated either with MI130110 or with naked anti-CD13 TEA1/8 mAb. Staining of CD13 (red), α-tubulin and acetylated α-tubulin (purple), β-tubulin (green) and chromosomes (blue), and a fluorescence merged image (merge) is shown. Scale bars are shown

The above results support the suitability of CD13 as an ADC target and thus encourage testing the biological activity of the ADC based on the anti-CD13 antibody. The in vitro anti-proliferative effect of MI130110 was evaluated against tumor cells expressing CD13 (HT1080, NB-4, and U-937) or not (Raji and RPMI 8226). The MI130110 ADC showed a clear anti-proliferative potential with remarkable selectivity for CD13-expressing cells (Table 1 and Fig. 3a), and both features were exclusive of the ADC molecule since PM050489 showed potent activity but not selectivity (Supplementary Table 1), whereas the naked TEA1/8 mAb did not cause any effect on the growth of any of the tested cell lines up to the highest concentration tested (1 μg/mL). Therefore, MI130110 combines the antitumor potential of the PM050489 payload with the selectivity of the anti-CD13 TEA1/8 mAb.

**Table 1:** Anti-proliferative activity of MI130110 in CD13-positive and negative cell lines. Values represent the geometric mean of three or more different experiments, each performed in triplicate

Tumor cell line	Cancer type	CD13 status	IC50 (μg/mL)	GSD
HT1080	Fibrosarcoma	Positive	0.17	1.32
NB-4	Leukemia	Positive	0.10	3.5
U-937	Lymphoma	Positive	0.14	3.3
RPMI 8226	Myeloma	Negative	> 1.0	-
Raji	Lymphoma	Negative	> 1.0	-

. *IC50* concentration that inhibits cell growth by 50%, *GSD* geometric standard deviation

To further endorse CD13 as a valid ADC target, we decided to confirm the appropriate intracellular processing of the MI130110-CD13 complex by interrogating whether the ADC payload was responsible for the anti-proliferative activity of MI130110, thus demonstrating an adequate intracellular payload release. The chemical class represented by PM050489 (including its dechlorinated analog, plocabulin, which is currently undergoing clinical trials for solid tumors) is known to bind tubulin tightly, thus impairing microtubule dynamics and cell division which will eventually result in cell death [31]. Therefore, the effect of the ADC on cell cycle was investigated. The flow cytometry data presented in Fig. 3b show that MI130110 arrested the cell cycle of fibrosarcoma HT1080 cells in G2 in a concentration-dependent manner since, according to the ratio of the peak areas, the percentage of cells in G2 rose from basal levels in the absence of ADC (22% at 24 h and 19% at 48 h) to 34% (24 h) or 31% (48 h) at 1 μg/mL to 54% (24 and 48 h) at 10 μg/mL. In contrast, MI130110 did not induce any effect on the cell cycle of the CD13-negative non-tumor endothelial EA.hy926 cells as the percentage of cells in G2 did not change with time (24 or 48 h) in the presence or in the absence of the ADC concentrations under study (1 μg/mL and 10 μg/mL), remaining at basal levels (Fig. 3b, right hand panels). Furthermore, fluorescence microscopy images of HT1080 cells treated with MI130110 for 24 h show the accumulation of cells undergoing mitosis, as indicated by chromosome condensation and spindle formation (identified by bright β-tubulin staining) (Fig. 3C). The percentage of cells undergoing and/or arrested in mitosis in MI130110-treated HT1080 cells was 46%, while in cells treated with anti-CD13 TEA1/8 mAb only 6.5% of them were dividing (Fig. 3d). This result is consistent with that obtained in cell cycle analysis (Fig. 3b) and is in agreement with the mechanism of action of the payload. In addition, dividing TEA1/8 mAb-treated cells could be found at different stages of mitosis (Fig. 3c, see arrows) while dividing MI130110-treated cells seem to be arrested at the earlier stages of mitosis. A more detailed analysis of the effect of MI130110 in mitosis demonstrated that chromosomes were fully condensed and that the nuclear membrane was disintegrated (Suppl Fig. 3), centrioles have moved, and the spindle formation was initiated (Fig. 3e). However, MI130110 treatment caused striking microtubule misalignment and mitotic spindle disarray, including frequent multipolar spindles, and the chromosomes failed to align in the equatorial plane (Fig. 3e). The failure of dividing cells to progress from methaphase would eventually result in mitotic catastrophe-mediated cell death. In contrast, none of these abnormalities was observed in anti-CD13 TEA1/8 mAb-treated cells, which could form normal spindles and aligned chromosomes and successfully completed mitosis (Fig. 3e).

It is noteworthy that, as shown in Fig. 3e, CD13 was endocyted and found in cytosolic vesicles away from the mitotic apparatus, thus further confirming that the payload was successfully released from the ADC.

**Fig. 4:**
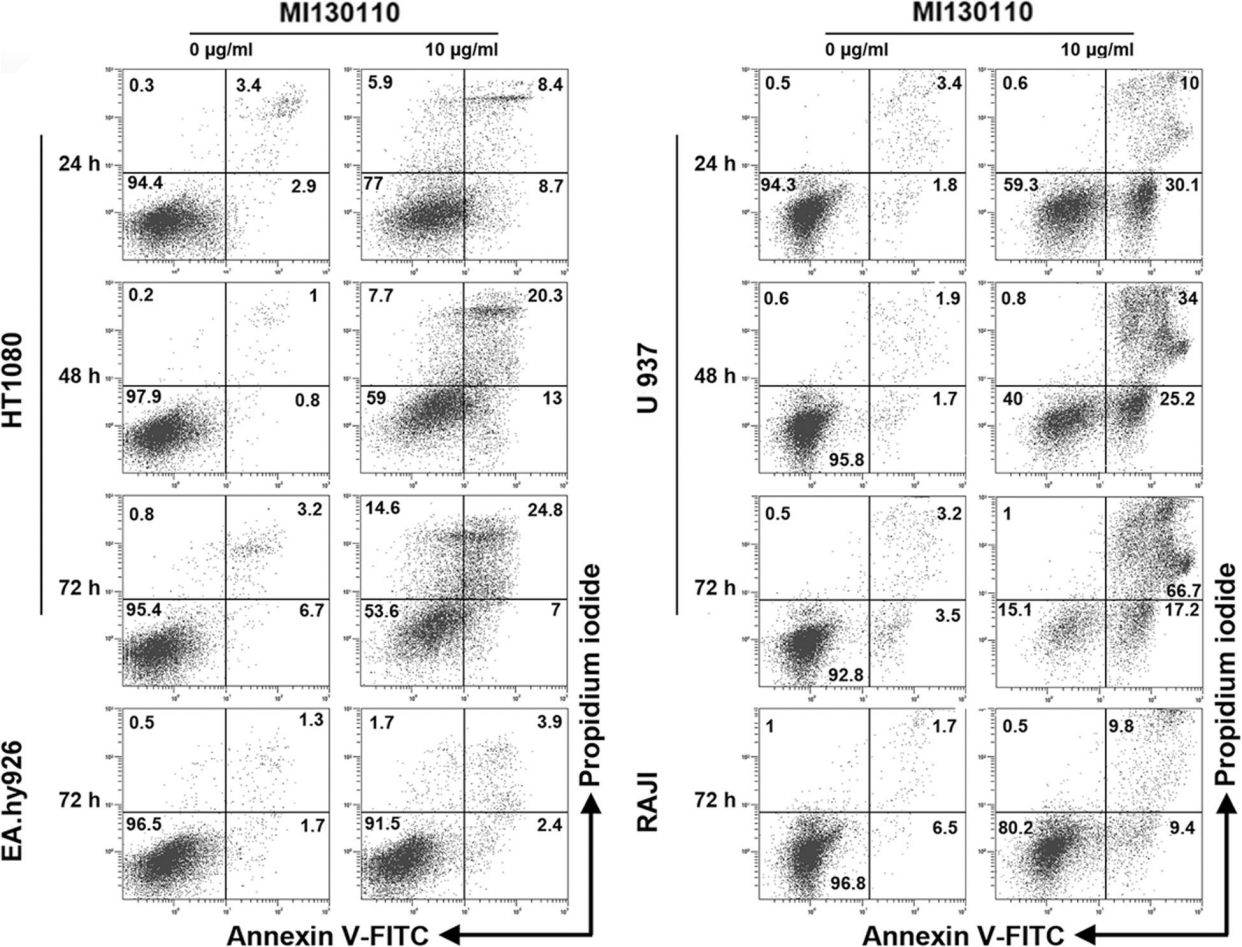
Effects of MI130110 on cell death. HT1080, U-937, EA.hy926, and Raji cells were cultured in the absence or in the presence of 10 μg/mL MI130110. Cells were analyzed by flow cytometry and representative dot plots of the annexin V- FITC and PI staining at the indicated times are shown. Figures show the percentage of cells in every quadrant

Accordingly, treatment with 10 μg/mL MI130110 seems to induce cell death only in CD13-expressing cells (HT1080 and U-937) but not in CD13-null cells (EA.hy926 and Raji) according to the flow cytometry experiment shown in Fig. 4. Of note, the fluorescent signal due to annexin V-FITC preceded that of PI in U-937 cells, thus suggesting apoptosis, but annexin V and PI staining were instead simultaneous in HT1080 which is suggestive of necrosis, two types of cell death that can occur after a mitotic catastrophe [32]. In contrast, only residual hints of cell death could be observed in the cell lines not expressing CD13. Together, these results demonstrate that the selective, CD13-dependent cytotoxic effect of MI130110 is exclusively due to its payload and it, in fact, demonstrates the proper intracellular processing of the ADC-antigen complex, thus reinforcing the role of CD13 as a suitable ADC target.

### In vivo effects of MI130110

**Fig. 5:**
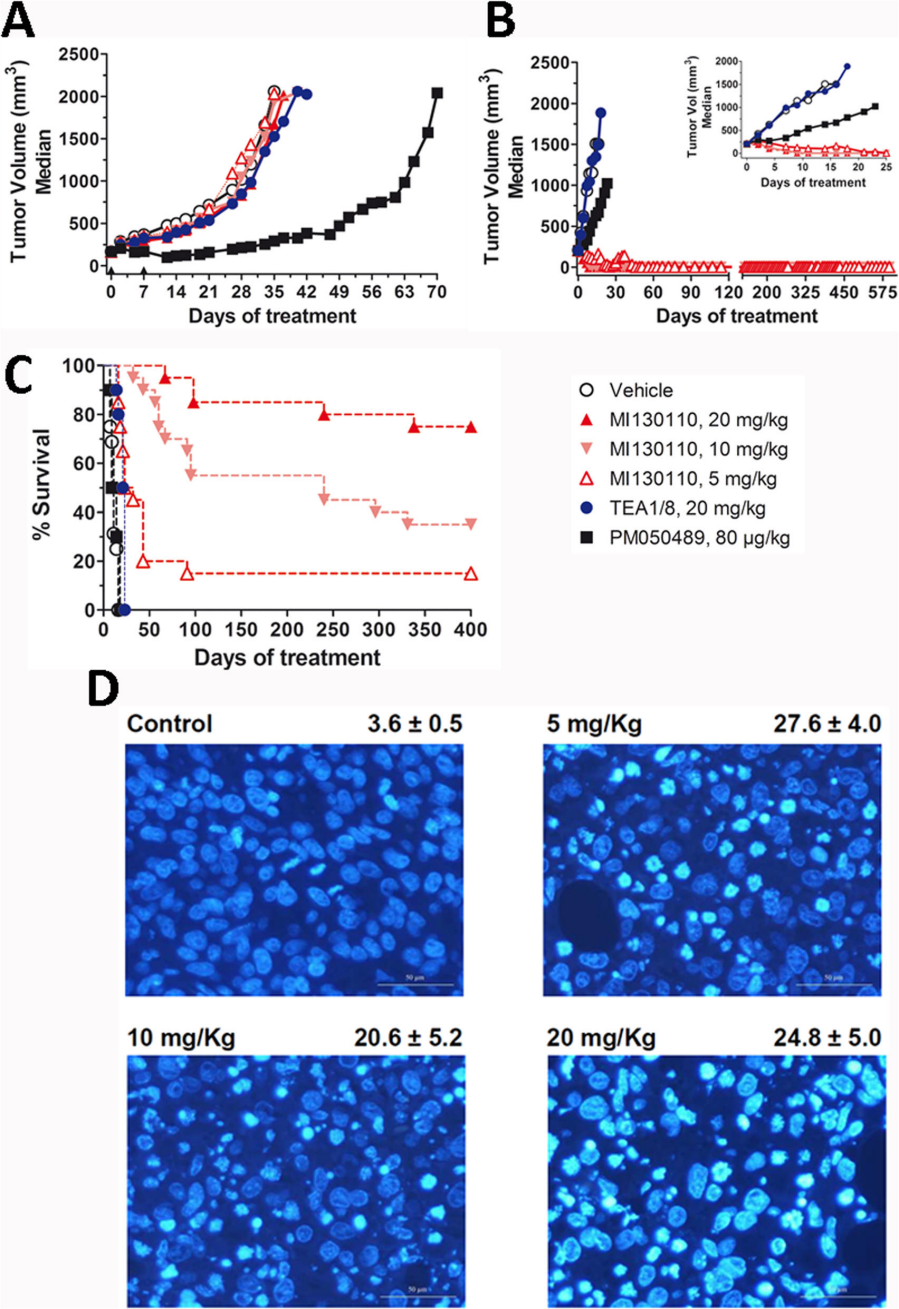
Biological effects of MI130110 in vivo. Animals (8–20 per group) were treated weekly for five consecutive weeks with either MI130110 at 5 (hollow triangles), 10 (pink inverted triangles), or 20 (red triangles) mg/kg, TEA1/8 at 20 mg/kg (blue circles), PM050489 at 80 μg/kg (black squares) or vehicle (hollow circles) following the procedure described in the “Materials and methods” section in mice xenografted with RPMI 8226 (**a**) or HT1080 (**b** and **c**) tumor cells. a and b show tumor growth evolution (with inset in b detailing HT1080 tumor evolution during the first 25 days), and c shows Kaplan-Meier survival curves for HT1080 xenografted animals. **d** Immunofluorescence analysis of tumor samples, withdrawn 24 h after the first treatment from mice xenografted with HT1080 tumor cells and treated with vehicle or with 5, 10, or 20 mg/kg MI130110, showing DNA staining with Hoechst 33258. Figures shown on the right hand side of each panel indicate the average (plus and minus the standard deviation) of the number of mitotic catastrophe nuclei per five high-power fields, with magnification being × 40

Based on this in vitro evidence of the potential antitumor effect of the CD13-targeting ADC, we next investigated whether it could be translated to an in vivo setting using mouse xenograft models of human tumors expressing CD13. The efficacy of the molecule was tested in xenografts models of fibrosarcoma (HT1080 cells) or myeloma (RPMI 8226 cells) expressing or not CD13, respectively. CD13 expression levels in cells from these tumors were confirmed by flow cytometry 24 h after the first administration to check that such expression had not been altered after tumor implantation (Suppl. Fig. 5). At the drug doses used in the experiment, no significant toxicity or body weight loss was observed in the treated animals. As shown in Fig. 5a, MI130110 up to 20 mg/kg did not elicit any antitumor activity in the RPMI 8226 myeloma xenograft model due to the negligible expression of CD13 by these cells (Fig. 1b and Suppl. Fig 5). Since PM050489 showed a potent effect in this model at a very low dose (80 μg/kg), the lack of response to the ADC treatment demonstrates that there is no spontaneous release of the payload in blood. However, as observed in Fig. 5b, MI130110 induced a strong antitumor response in the CD13-expressing HT1080 fibrosarcoma model. Animals treated with 5, 10, or 20 mg/kg experienced complete tumor remissions during the treatment (Fig. 5b and Suppl. Fig. 5). Hence, 8 out of 20 animals treated with MI130110 at 5 mg/kg experienced complete remissions from days 14 to 28, and 3 out of 20 animals in this group remained in tumor remission beyond day 390. All animals treated with MI130110 at 10 mg/kg experienced complete remissions from days 9 to 21, and 11 out of 20 animals in this group still had tumor remission beyond day 230. Finally, all animals treated with MI130110 at the highest dose (20 mg/kg), experienced complete remissions from days 7 to 28, and 17 out of 20 animals in this group were tumor-free beyond day 330. Likewise, PM050489 also induced anti-tumoral activity in animals treated with this compound at 0.075 mg/kg (Fig. 5b), although systemic toxicity was observed, and therefore treatments were discontinued and animals sacrificed after day 23. On the other hand, treatment with the anti-CD13 TEA1/8 mAb did not cause any response. Survival curves demonstrated that treatment with MI130110 at 5, 10, or 20 mg/kg increased the survival time with statistically significant differences regarding the placebo-, PM050489-, or anti-CD13-treated animals (Fig. 5c). Remarkably, nuclear staining of HT1080 tumor samples from animals treated with MI130110 at any dose showed an increase in the number of mitotic catastrophes significantly higher than those observed in samples from the placebo-treated group (Fig. 5d), which is consistent with the mechanism of action of PM050489, hence confirming that the antitumor response caused by the ADC in vivo is due to the activity of its payload. Altogether, these results clearly demonstrate that the anti-CD13-based ADC MI130110 is endowed with extraordinary antitumor potential both in vitro and in vivo, and therefore CD13 can be deemed as a promising novel target for the development of ADCs for anticancer therapy.

## Discussion

Being an emergent drug class, antibody-drug conjugates are widely considered as an attractive opportunity to improve the selectivity of cancer therapies. The successful cases of the four ADCs approved by the FDA to date [18] have fostered the search of novel entities endowed with similar beneficial properties. The nature of the antibody target constitutes the keystone for selectivity which, by itself, constitutes the most innovative and valuable feature of ADCs. Not surprisingly, the search for novel ADC targets is one of the most compelling areas of research in the field.

CD13 expression is fairly restricted to the myeloid lineage. Although CD13 depositions are also found in the luminal region of the digestive system and on the apical zone of cells conforming the efferent conducts of some digestive glands, prostate, and on renal tubules (www.proteinatlas.org), this apical CD13 expression of luminal epithelial cells should not be readily accessible by the ADC. Regarding CD13 expression in tumors, CD13 mRNA and protein seems to be abnormally overexpressed, i.e., in some samples of the melanoma, glioma, lung, liver, pancreatic, stomach, renal, prostate, testis, endometrial, and ovarian cancers [33, 34] (data from The Human Protein Atlas available at www.proteinatlas.org). Indeed, there is a very active research on tumor stratification based on CD13 expression, and there are many reports in the literature indicating that CD13 expression is an unfavorable prognosis factor in a variety of cancers. Those tumors include pancreas [10] and colon cancers [11], non-small cell lung cancer [12, 13], malignant pleural mesothelioma [14], hepatoblastoma [15], hepatocellular carcinoma [35], clear cell papillary renal cell carcinoma [36], scirrhous gastric cancer [37], lymphoplasmatic lymphoma [38], and soft tissue sarcoma [16]. Some of these tumors are rare and/or have poor prognosis, and new therapeutic approaches are urgently needed to increase patient’s survival.

In addition, CD13-targeted therapies would likely broadly benefit patients with various types of myeloid malignancies [39], in particular for those with lower survival rate, such as acute myeloid leukemia, the most common acute leukemia in adults, with 17% survival rate at 5 years. Myelodysplastic and myeloproliferative neoplasms, including chronic myeloid leukemia, with an overall 20% 5-year survival rate [40] are also potential targets of this ADC.

Furthermore, CD13 is also found in vascular endothelium surrounding tumors [7] (Suppl. Fig. 1), where it plays a critical role in angiogenesis [4]. This differential expression pattern with respect to healthy tissues makes of CD13 an attractive target for the selective delivery of drugs or cytokines. Indeed, a CD13-specific single monomeric variable antibody domain tagged to tumor necrosis factor (TNF) or interferon (IFN)-γ has been used to kill tumors by targeting the tumor neovasculature [41]. In addition, peptides based in the NGR motif, found out in a phage display exercise to selectively bind CD13 [42], have already been tested in “tumor homing” strategies for chemotherapeutic agents (compiled in [3]) directed towards CD13-expressing cells. Such peptide sequence was used for conjugation with drugs like doxorubicin [43], cisplatin [44], and lidamycin [45] in experimental preclinical models, and conjugates to human TNF [46] or tissue factor [47] have even entered clinical trials. Likewise, such sequence has been used for the preparation of a prodrug of melphalan (melflufen or “J1”, [3]) which is currently undergoing clinical trials for multiple myeloma. Of note is that a single CD13-specific single-chain V-Ig-fragment (scFv13) linked to exotoxin A from *Pseudomonas aeruginosa* has been probed to inhibit proliferation of human cancer cell lines in vitro [48]. However, development of standard ADC targeting CD13 has been neglected. This might have been the result of the availability of alternative approaches to target CD13 that would not require mAb humanization, as described above, but it might have also been influenced by the limited available information regarding the internalization efficiency of the antibody-CD13 complex and its subsequent intracellular processing. Our results demonstrate that the monoclonal TEA1/8 antibody is readily internalized upon interaction with its target in CD13-expressing cells like HT1080, NB-4, and U-937, with more than 50% internalization rate after 3 h, and this efficiency remains unaltered after conjugation with PM050489. The internalization rates that can be inferred from these results are comparable to those described for other ADCs. Indeed, when compared to the values published for benchmarking ADCs and antibodies, the internalization rates of TEA1/8 mAb and MI130110 are faster than those reported for trastuzumab alone [49, 50], maytansinoid-trastuzumab conjugates [51], or brentuximab vedotin [52], and it is in the same range than the internalization rate described for gemtuzumab ozogamicin [53, 54]. Of note is that the reported internalization rates of the anti-epidermal growth factor receptor (EGFR) antibody Ab033 are about 10-fold faster than those reported for the above mentioned conjugates [55]. However, it is commonly accepted that an excessively fast cellular uptake may jeopardize the therapeutic efficiency of the ADC by hampering tumor penetration [56]. Therefore, the intermediate internalization rates shown by TEA1/8 and MI130110, in a similar range to that of Mylotarg, seem to be adequate for the intended use.

Likewise, the moderately high affinity (circa 2 nM) shown by TEA1/8 and MI130110 is certainly weaker than that observed for other ADC-related antibody-antigen pairs falling in the pM scale like gemtuzumab [57], but in a similar range to that described for trastuzumab [58, 59] or brentuximab [60]. As explained above regarding cellular uptake, extremely high affinities usually lead to low therapeutic efficacies [61], as they are associated with slow off-rates [62], hence deficient ADC release and poor intracellular processing as well as impaired tumor penetration [63]. Consequently, the moderately high affinity of TEA1/8 mAb for CD13, matching those of the Kadcyla and Adcetris antibodies for their targets, together with the mild internalization rate similar to that of Mylotarg, endorse CD13 as a suitable ADC target and TEA1/8 mAb as a valid antibody for conjugation purposes.

The antitumor properties exhibited by MI130110 in vitro and in vivo, with the excellent results obtained in the murine xenograft models, support these arguments. MI130110 caused a remarkable antitumor effect in the fibrosarcoma HT1080 model leading to complete tumor remissions that lasted beyond 1 year in a significant proportion of the treated animals. The lack of activity observed in the CD13-negative RPMI 8226 myeloma model demonstrates that payload release does not occur spontaneously, while the appearance of mitotic catastrophes (consistent with the mechanism of action of the PM050489 payload) in HT1080 tumor cells observed both in vitro and in tumors from xenografted animals that had been treated with MI13010 indicates an adequate intracellular processing of the ADC in CD13-expressing cells, therefore confirming that conjugation to TEA1/8 mAb does not hinder the anti-proliferative properties of the marine drug.

Finally, it is important to mention that secondary or acquired resistance to anti-mitotic drugs is often related to the upregulation membrane efflux pumps of the ATP-binding cassette (ABC) family that actively exports out of the cell the cytotoxic compounds [64, 65]. By using the MI130110 ADC and, therefore, altering the entry pathway of the anti-mitotic PM050489 drug to the cell, the efficacy of the ABC family might be reduced, allowing the drug to be effective in tumor cells with this type of resistance.

In overall, the results presented in this study demonstrate that CD13 is a suitable target for the development of novel ADCs of promising therapeutic potential in the fight against cancers of different origin and poor prognostic.

## Conclusions

In this report, we have described for the first time the generation of an anti-CD13 mAb-based ADC, MI130110. Our results on the specificity and activity of MI130110, both in vitro and in vivo demonstrate that it combines the strong antitumor activity of the PM050489 payload with the selectivity of the anti-CD13 TEA1/8 mAb and have confirmed the correct intracellular processing of the ADC. Altogether, our results demonstrate the suitability of CD13 as a novel ADC target and the effectiveness of MI130110 as a promising antitumor therapeutic agent.

## Supplementary information

**Additional file 1: **Supplementary Materials and Methods, **Table S1.** Anti-proliferative activity of PM050489 in CD13 positive and negative cell lines. **Figure S1.** Immunohistochemistry analysis of CD13 expression in tumor samples. **Figure S2.** Chromatography analysis of TEA1/8 mAb and MI130110. **Figure S3.** Immunofluorescence analysis of the nuclear membrane and chromosome condensation. **Figure S4.** Flow cytometry analysis of cells used in xenograft models. **Figure S4.** Evolution of HT1080 tumor volumes at 4 given days. **Figure S4.** Evolution of HT1080 tumor volumes at 4 given days in MI130110-treated xenografted mice.

## Data Availability

The datasets used and/or analyzed during the current study are available from the corresponding authors on reasonable request.

## Abbreviations

ADC: Antibody-drug conjugate
ANPEP: Alanyl aminopeptidase
APN: Aminopeptidase N
BSA: Bovine serum albumin
CR: Complete tumor regression
DAPI: 4′,6-Diamidino-2-phenylindole
DMSO: Dimethyl sulfoxide
EDTA: Ethylenediaminetetraacetic acid
EGFR: Epidermal growth factor receptor
FBS: Fetal bovine serum
FDA: Food and Drug Administration
FITC: Fluorescein isotiocyanate
HIC: Hydrophobic interactions chromatography
IFN-γ: Interferon gamma
mAb: Monoclonal antibody
MFI: Mean fluorescence intensity
MTT: 3-(4,5-dimethylthiazol-2-yl)-2,5-diphenyltetrazolium bromide
NGR: Asn-Gly-Arg
PBS: Phosphate buffered saline
PI: Propidium iodide
TCEP: Tris(2-carboxyethyl) phosphine hydrochloride
TNF: Tumor necrosis factor
