# Supplementary material for: CD13 as a new tumor target for antibody-drug conjugates: validation with the conjugate MI130110
Source: J Hematol Oncol. 2020 Apr 7;13:32. doi: 10.1186/s13045-020-00865-7 (PMC7140356; doi:10.1186/s13045-020-00865-7)
Supplement: Supplementary file 1 — Additional file 1: Supplementary Materials and Methods, Table S1. Anti-proliferative activity of PM050489 in CD13 positive and negative cell lines. Figure S1. Immunohistochemistry analysis of CD13 expression in tumor samples. Figure S2. Chromatography analysis of TEA1/8 mAb and MI130110. Figure S3. Immunofluorescence analysis of the nuclear membrane and chromosome condensation. Figure S4. Flow cytometry analysis of cells used in xenograft models. Figure S4. Evolution of HT1080 tumor volumes at 4 given days. Figure S4. Evolution of HT1080 tumor volumes at 4 given days in MI130110-treated xenografted mice. [file 13045_2020_865_MOESM1_ESM.docx]

**SUPPLEMENTARY INFORMATION**

**Dominguez el al. CD13 AS A NEW TUMOR TARGET FOR ANTIBODY-DRUG CONJUGATES: VALIDATION WITH THE CONJUGATE MI130110**

**ADDITIONAL FILE 1**

**SUPPLEMENTARY MATERIALS AND METHODS**

Cell lines and cell culture

The following human cell lines were obtained from the American Type Culture Collection (ATCC) (Manassas, VA, USA): U-937 (CRL-1593.2™), histiocytic lymphoma; HT1080 (CCL-121™), fibrosarcoma; Raji (CCL-86™) Burkitt's lymphoma; RPMI 8226 (CRM-CCL-155™), myeloma; and EA.hy926 (CRL-2922™), endothelial. The human acute promyelocytic leukemia cell line NB‑4 (ACC 207) was obtained from the Leibniz-Institut DSMZ (Braunschweig, Germany). Cell lines were maintained in either EMEM (HT1080), DMEM (EA.hy926) or RPMI-1640 (rest of the lines) media supplemented with 10% fetal bovine serum (FBS), 2 mM L-glutamine, 100 U/mL penicillin and 100 µg/mL streptomycin at 37ºC and 5% CO2.

**Preparation of the anti-CD13 TEA1/8 monoclonal antibody**.

BALB/c mice were immunized by injecting 1.5E07 human endothelial cells intraperitoneally on days −45 and −30 and intravenously on day −3. On day 0 spleens from these animals were removed and spleen cells were fused with SP2 mouse myeloma cells at a ratio of 4:1 according to standard techniques to produce the corresponding hybridomas and distributed on 96-well tissue culture plates (Costar Corp., Cambridge, MA). After 2 weeks, hybridoma culture supernatants were harvested and their reactivity against the cell line used in the immunization step was tested by flow cytometry. Positive supernatants were assayed by immunofluorescence staining of human endothelial cells used as antigens. Hybridomas showing a specific staining, immunoprecipitation pattern and cell distribution were selected and cloned and subcloned by limiting dilution. Once the clones were selected, cells were cultured in RPMI-1640 medium supplemented with 10% (v/v) fetal calf serum, 2 mM L-glutamine, 100 U/mL penicillin and 100 µg/mL streptomycin at 37ºC during 3-4 days until the medium turned pale yellow. At that point, two thirds of the medium volume were removed, centrifuged at 1,000 x g for 10 min to pellet the cells and the supernatant was either centrifuged again for further cleaning at 3,000 x g for 10 min or filtered through 22 µm pore size membranes. The clarified supernatant was subjected to precipitation with 55% saturation ammonium sulphate and the resulting pellet was resuspended in 100 mM Tris-HCl pH 7.8 (1 mL per 100 mL of the original clarified supernatant) and dialyzed at 4ºC for 16-24 h against 5 L of 100 mM Tris-HCl pH 7.8 with 150 mM NaCl, changing the dialyzing solution at least three times. The dialyzed material was finally loaded onto a Protein A-Sepharose column and the monoclonal antibody was eluted with 100 mM sodium citrate pH 3.0 or alternatively with 1 M glycine pH 3.0. Those fractions containing the antibody were neutralized with 2 M Tris-HCl pH 9.0 and finally dialyzed against phosphate buffered saline (PBS) and stored at ‑80ºC until its use.

Preparation and analysis of MI130110

A 70 µM (10.5 mg/mL) solution of the TEA1/8 mAb in 50 mM sodium phosphate pH 8.0 buffer was mixed with the appropriate amount of a 5 mM solution of Tris(2-carboxyethyl) phosphine hydrochloride (TCEP) in water to keep the reducing agent in a 2.5-fold molar excess over the antibody. The mixture was incubated and stirred for 60 min at 20ºC and afterwards the appropriate volume of a 10 mM PM120160 solution in dimethyl sulfoxide (DMSO) was added to reach a fair molar excess of the compound over the antibody. DMSO was added if needed to keep its concentration at 5% (v/v) and the mixture was incubated for 30 min at 20ºC. Afterwards N-Acetyl-cysteine was added to quench the reaction, using the appropriate volume of a 10 mM solution in water to match the concentration of PM120160. The resulting ADC was finally purified from the rest of reagents by gel filtration in Sephadex G-25 using PD-10 columns. The presence of aggregates was checked by analytical size exclusion chromatography using an Äkta FPLC system equipped with a Superdex-100 10/300 column running an isocratic method with PBS at 1 mL/min: if the area of the peak corresponding to aggregates exceeded 10% of the total peak area, monomers were purified using the same chromatography system with a Superdex 200 16/600 preparative column running the same method described above. Final ADC concentration was spectrophotometrically determined and if the ADC concentration was below 2 mg/mL it was concentrated using Vivaspin devices from GE Healthcare and the new concentration was again determined as above.

Analysis of MI130110 by hydrophobic interactions chromatography (HIC) was performed on a Agilent 1100 HPLC system (Agilent, Santa Clara, CA). equipped with a 3.5 x 4.6 mm TSK-gel butyl-NPR column with 2.5 µm particle size (Tosoh Bioscience, Tokyo, Japan), using 1.5 M ammonium sulfate in 25 mM sodium phosphate, pH 7.5 as mobile phase A and 25 % (v/v) isopropanol in 25 mM sodium phosphate pH 7.5 as mobile phase B. Elution was run at a constant 0.8 mL/min flow rate using a 0-100% B gradient in 12 min followed by a 5-min isocratic elution in 100% B.

**Cell isolation from xenografted tumors**

Tumors from mouse xenografts were excised and washed with PBS and then cut in small pieces with a blade. Tumors were then incubated with 0.2 U/mL liberase (Sigma-Aldrich) for 50 minutes at 37 ºC with continuous shaking. Supernatant was collected and supplemented with 1/5 v/v of PBS containing 10 mM Ethylenediaminetetraacetic acid (EDTA) and 0.5 % (w/v) BSA and then further mechanically disrupted by passing through a 19G needle. The resulting cell suspension was filtered using a 50 μm cell strainer and centrifuged at 400 x g for 5 min. Cells were washed twice with PBS containing 2 mM EDTA and 0.1% BSA and analyzed for CD13 expression by flow cytometry, as described in Materials and Methods.

Immunohistochemistry analysis of mouse and human tumor samples

Mouse tumors (or skin around the former tumor site) from xenografted mice were dissected, formalin-fixed and paraffin-embedded for histopathology evaluations. Tissue sections (5 μm) were stained with anti-CD13 TEA1/8 mAb and color was developed using a diaminobenzidine-based detection method (Vector Laboratories, Burlingame, CA) and mounted in DPX (Fluka).

CD13 immunostaining of human breast cancer samples was performed in tissue samples from metastatic breast cancer patients enrolled in the ET-B-027-06 Phase II clinical trial sponsored by PharmaMar (EudraCT Number: 2007-000794-31). Immunohistochemical staining of breast samples was performed at MorningStar (Lake Forest, CA) using CD13 Cell Marque rabbit monoclonal antibody (Sigma, St Louis, MO, catalog number 113R-16) and MACH 4 Universal HRP-Polymer with diaminobenzidine (Biocare Medical, Pacheco, CA), counterstaining was performed with Mayer’s Hematoxylin. The immunostaining process was completed using a Dako Autostainer Plus model LV-1 instrument. Immunohistochemical staining of archival paraffin blocks with representative types of tumors from patients from Hospital de la Princesa, including acute myeloid leukemia, myeloid sarcoma and liposarcoma and leiomyosarcoma. Tissue sections (5 μm) were stained with anti-CD13 TEA1/8 mAb and color was developed using a diaminobenzidine-based detection method (Vector Laboratories, Burlingame, CA) and mounted in DPX (Fluka).

**ADDITIONAL FILE 2**

Table S1: Anti-proliferative activity of PM050489 in CD13-positive and CD13-negative cell lines. Values represent the geometric mean of three or more different experiments, each performed in triplicate. IC50, concentration that inhibits cell growth by 50%. GSD, geometric standard deviation.

| **Tumor cell line** | **Cancer type** | **CD13 status** | **IC50 (nM)** | **GSD** |
| --- | --- | --- | --- | --- |
| HT1080 | Fibrosarcoma | Positive | 0.40 | 1.46 |
| NB-4 | Leukemia | Positive | 0.04 | 1.71 |
| U-937 | Lymphoma | Positive | 0.09 | 1.48 |
| RPMI 8226 | Myeloma | Negative | 0.14 | 1.30 |
| Raji | Lymphoma | Negative | 0.36 | 1.59 |


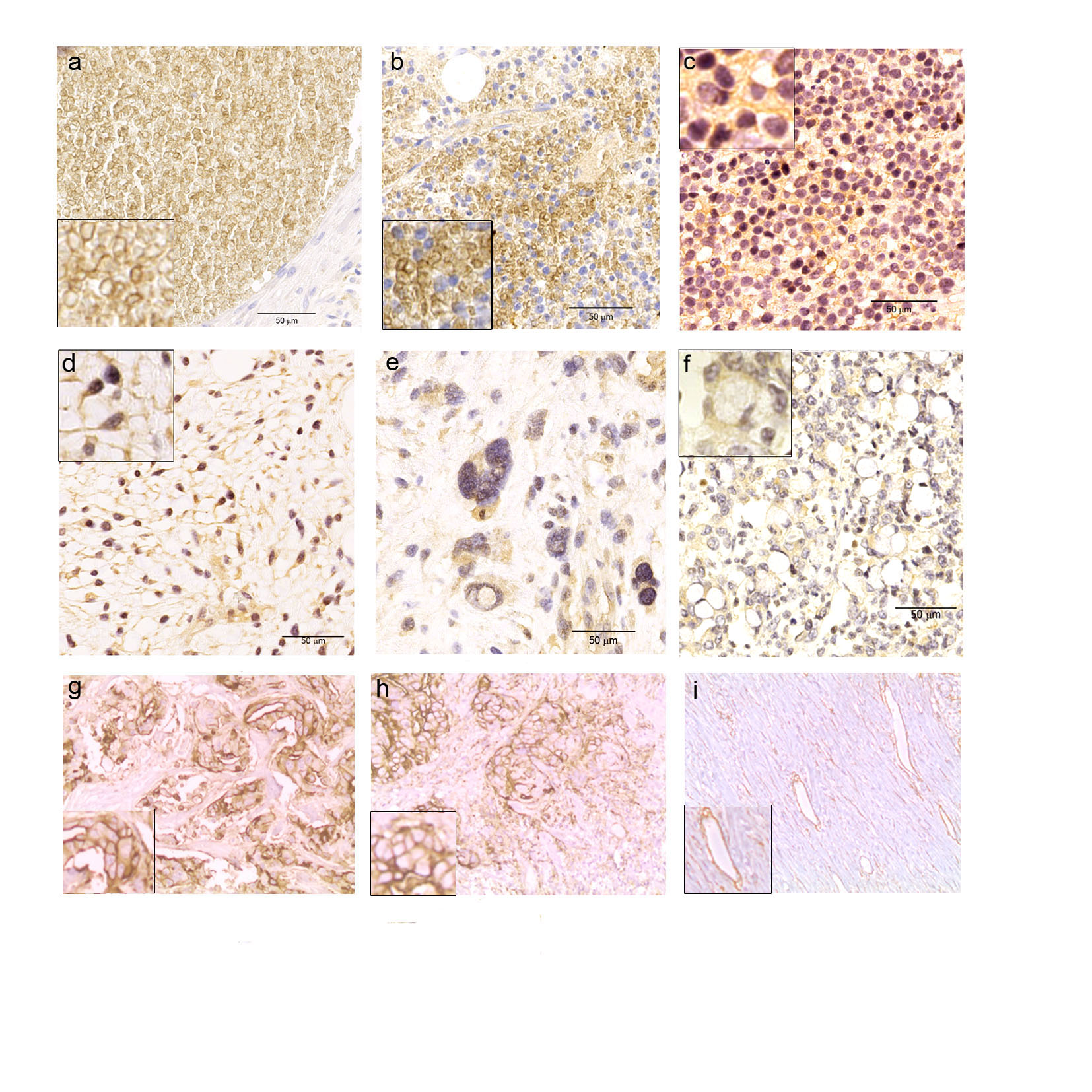


Figure S1: Immunohistochemistry analysis of CD13 expression in tumor samples from cancer patients. a and b: Acute myeloblastic leukemia c: myeloid sarcoma, d: well-differentiated liposarcoma, e: dedifferentiated liposarcoma, f: signet ring cell gastric adenocarcinoma, g and h: ductal breast carcinoma, i: breast carcinoma showing CD13-negative tumor cells but showing CD13 expression in tumor vessels. Higher magnification of representative cells is shown in some of the slides. Scale bar (50 µm) is shown.


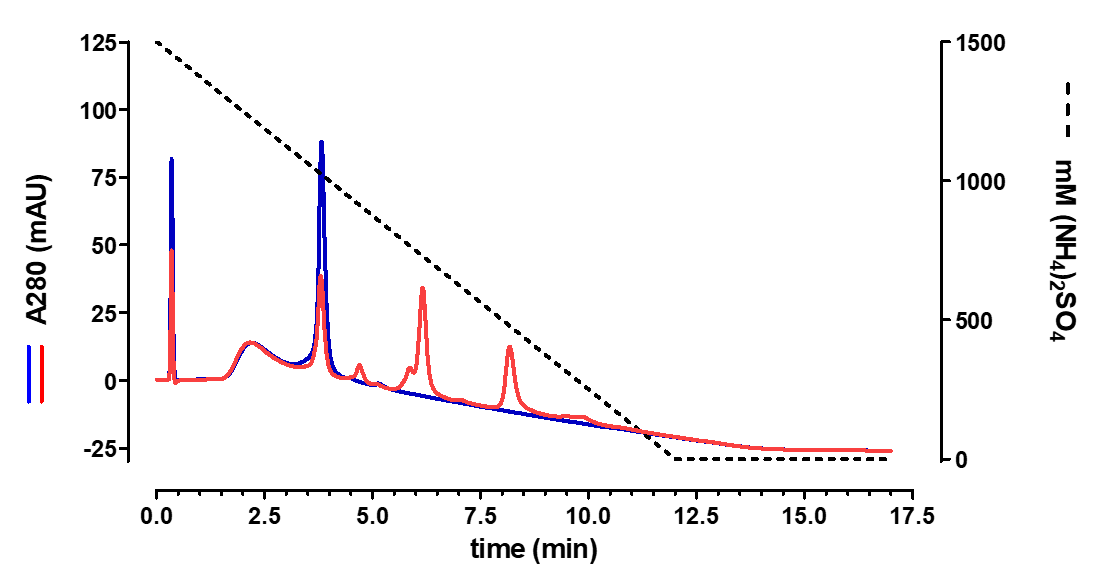


Figure S2: Chromatography analysis of TEA1/8 mAb and MI130110. HIC analysis of TEA1/8 mAb (blue line) and MI130110 (red line) run as described under Materials and Methods. The dotted black line shows the ammonium sulfate gradient. The peak at 3.8 min must correspond to the naked antibody, whereas peaks of higher hydrophobicity observed at 4.7, 6.2 and 8.2 min must correspond to conjugates of different payload stoichiometries as they are detected in the MI130110 sample but not in the TEA1/8 one.


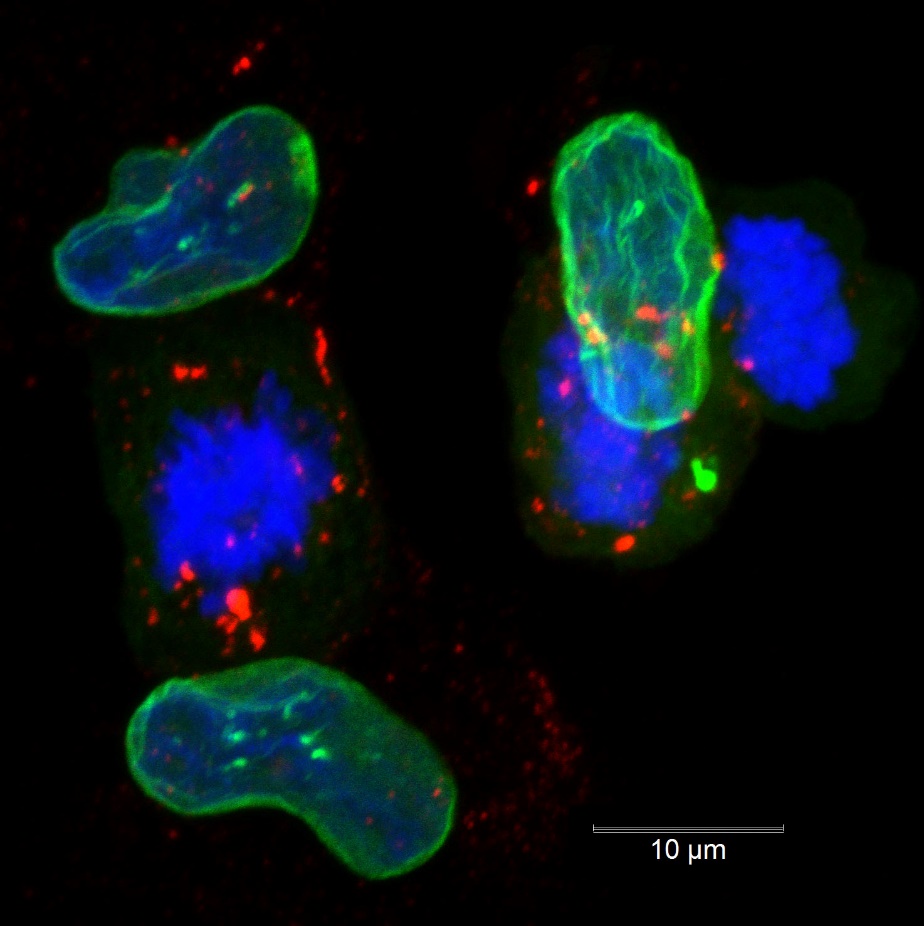


Figure S3: Immunofluorescence analysis of the nuclear membrane and chromosome condensation in cells treated with MI130110. HT1080 cells were treated with 5 μg/mL of MI130110 for 24 h at 37 ºC and then processed for immunofluorescence as described in Materials and Methods. Figure shows representative cells undergoing mitosis (x 128 optical magnification). Staining of CD13 (red), lamin A/C (green) and chromosomes (blue) are shown as a merged image. Scale bar (10 µm) is shown.


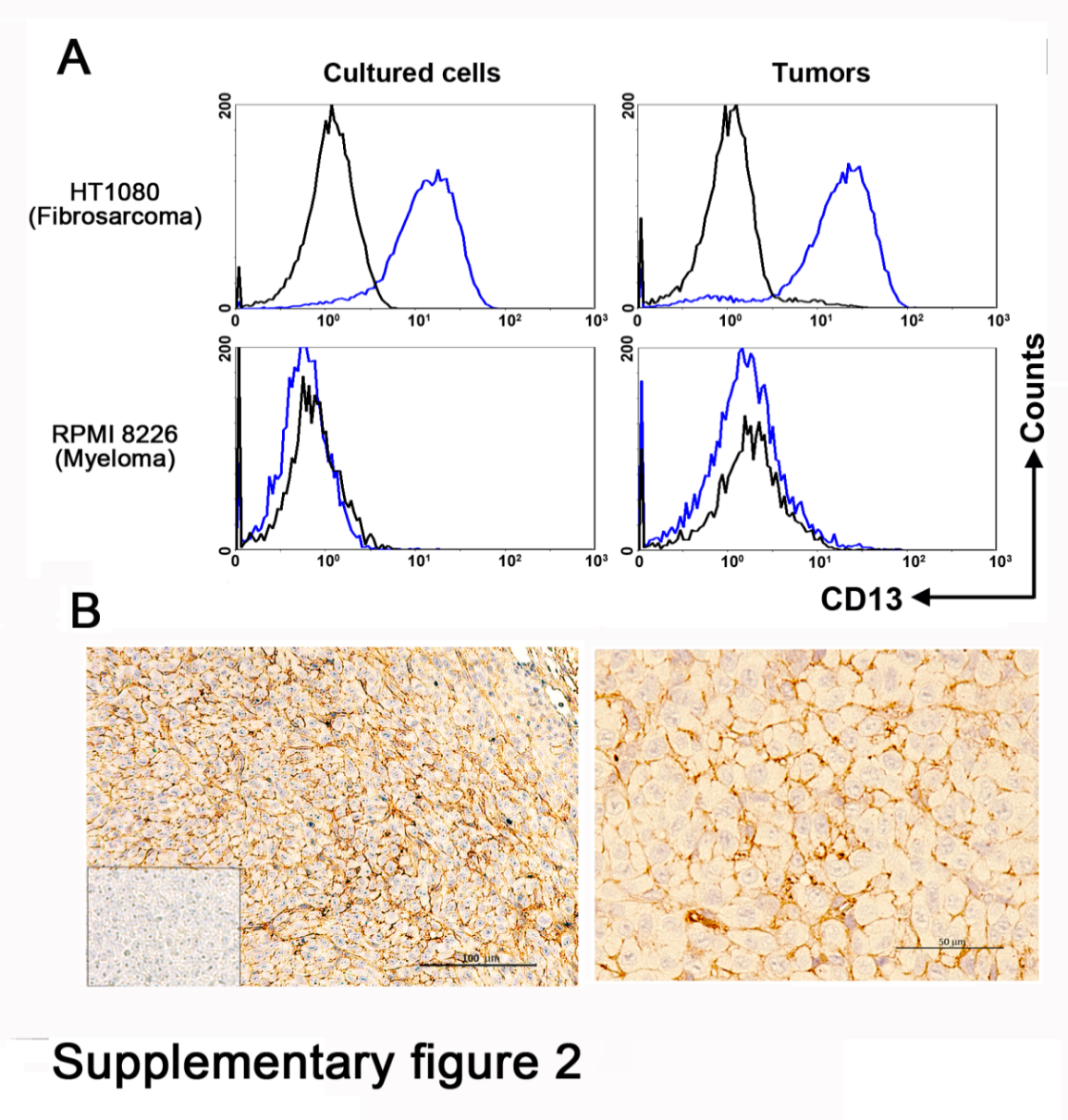


Figure S4: Flow cytometry analysis of cells used in xenograft models. A: The expression of CD13 in HT1080 and RPMI 8226 cell lines either in culture medium (left) or xenografted in mice (right) was assessed by flow cytometry (blue line). Isotype control staining is also shown (black line). Cells from tumors were mechanically and enzymatically disaggregated before being analyzed for CD13 expression by flow cytometry. B: Immunohistochemistry analysis. HT1080 tumors from xenografted mice were fixed and embedded in paraffin. CD13 expression was assessed by immunohistochemistry as described in Materials and Methods. Figure shows the staining of representative HT1080 tumor sections with TEA1/8 (top panel x200, Bottom panel, x400). The inset in the top panel represents a x200 photomicrograph of the tumor stained with an irrelevant IgG2 as control. Scale bars are shown.


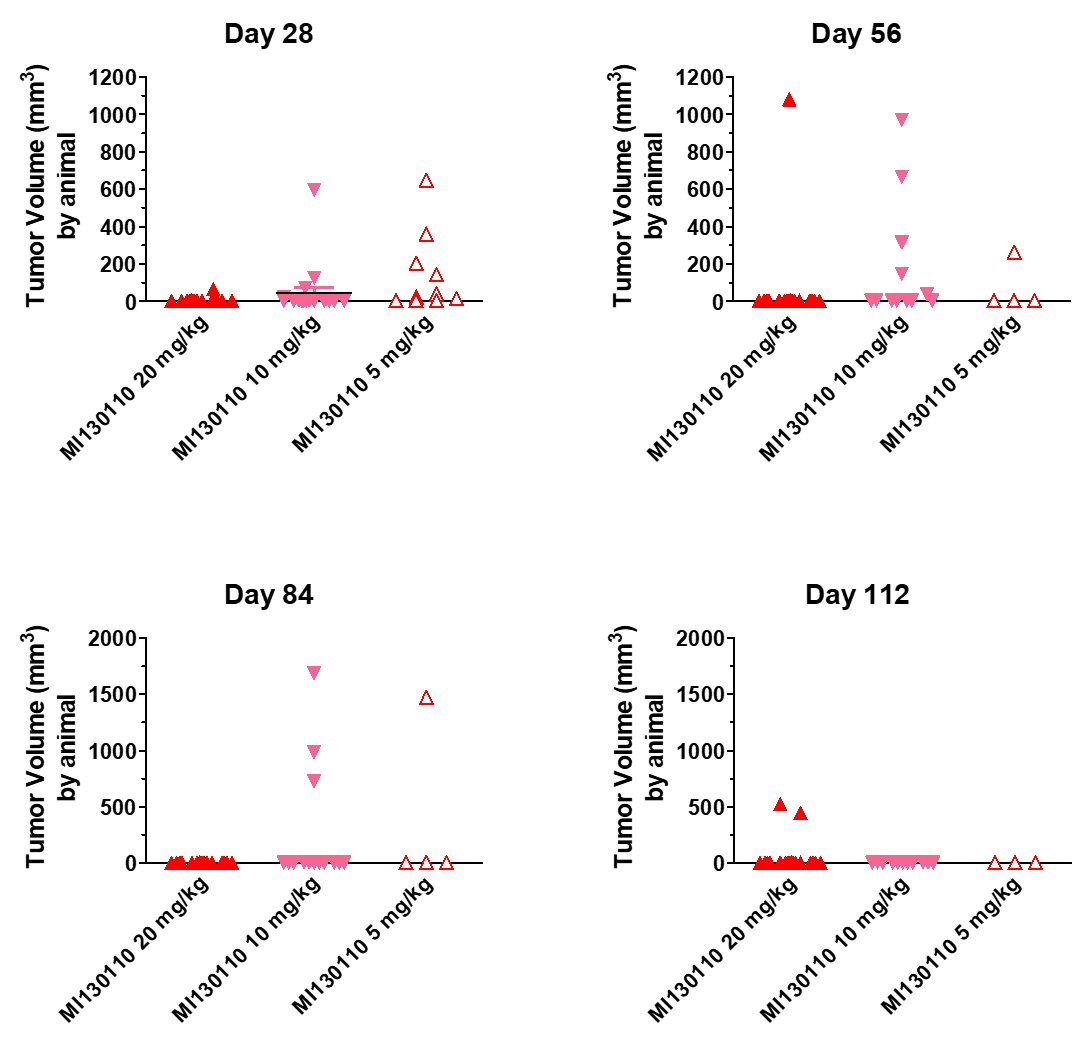


Figure S5: Evolution of HT1080 tumor volumes at 4 given days in MI130110-treated xenografted mice. Tumor models were grown and animals treated as detailed in the text, the graphs show tumor volumes for each mouse under study after 28, 56, 84 and 112 days of treatment.
